# The Role of Prostatitis in Prostate Cancer: Meta-Analysis

**DOI:** 10.1371/journal.pone.0085179

**Published:** 2013-12-31

**Authors:** Junyi Jiang, Jinyi Li, Zhang Yunxia, Hong Zhu, Junjiang Liu, Chris Pumill

**Affiliations:** 1 Public Health Institution, The Ohio State University, Columbus, Ohio, United States of America; 2 Department of Urology, Weill Cornell Medical College, New York, New York, United States of America; 3 Department of Urology, Hebei General Hospital, Shijiazhuang City, People’s Republic of China; 4 Rutgers Robert Wood Johnson Medical School, Rutgers University, New Brunswick, New Jersey, United States of America; Eberhard-Karls University, Germany

## Abstract

**Objective:**

Use systematic review methods to quantify the association between prostatitis and prostate cancer, under both fixed and random effects model.

**Evidence Acquisition:**

Case control studies of prostate cancer with information on prostatitis history. All studies published between 1990-2012, were collected to calculate a pooled odds ratio. Selection criteria: the selection criteria are as follows: human case control studies; published from May 1990 to July 2012; containing number of prostatitis, and prostate cancer cases.

**Evidence Synthesis:**

In total, 20 case control studies were included. A significant association between prostatitis and prostate cancer was found, under both fixed effect model (pooled OR=1.50, 95%CI: 1.39-1.62), and random effects model (OR=1.64, 95%CI: 1.36-1.98). Personal interview based case control studies showed a high level of association (fixed effect model: pooled OR=1.59, 95%CI: 1.47-1.73, random effects model: pooled OR= 1.87, 95%CI: 1.52-2.29), compared with clinical based studies (fixed effect model: pooled OR=1.05, 95%CI: 0.86-1.28, random effects model: pooled OR= 0.98, 95%CI: 0.67-1.45). Additionally, pooled ORs, were calculated for each decade. In a fixed effect model: 1990’s: OR=1.58, 95% CI: 1.35-1.84; 2000’s: OR=1.59, 95% CI: 1.40-1.79; 2010’s: OR=1.37, 95% CI: 1.22-1.56. In a random effects model: 1990’s: OR=1.98, 95% CI: 1.08-3.62; 2000’s: OR=1.64, 95% CI: 1.23-2.19; 2010’s: OR=1.34, 95% CI: 1.03-1.73. Finally a meta-analysis stratified by each country was conducted. In fixed effect models, U.S: pooled OR =1.45, 95%CI: 1.34-1.57; China: pooled OR =4.67, 95%CI: 3.08-7.07; Cuba: pooled OR =1.43, 95%CI: 1.00-2.04; Italy: pooled OR =0.61, 95%CI: 0.13-2.90. In random effects model, U.S: pooled OR=1.50, 95%CI: 1.25-1.80; China: pooled OR =4.67, 95%CI: 3.08-7.07; Cuba: pooled OR =1.43, 95%CI: 1.00-2.04; Italy: pooled OR =0.61, 95%CI: 0.13-2.90.CONCLUSIONS: the present meta-analysis provides the statistical evidence that the association between prostatitis and prostate cancer is significant.

## Introduction

Today, inflammation is present in approximately 17% of all cancer cases[1]. Rudolf Virchow was the first to find a positive association between inflammation and cancer in 1863[2]; finding a high density of leukocytes in neoplastic samples. After that, both epidemiological and biological studies focused the on function of inflammation in order to provide evidence of an association. Epidemiological studies, including case control and cohort studies, concluded that inflammation is highly correlated with several types of cancers including bowel, stomach, esophageal, etc [3–6]. Biological studies provided evidence that active oxygen and nitrogen radicals produced by inflammation tissue increased the risk of cancer by suppressing antitumor activity and stimulating carcinogenesis [7,8]. New genetic evidence suggests that transcription factors, NF-kB and STAT3 play a role in the association between inflammation and cancer [9,10].

Prostatitis, is defined as inflammation of the prostate gland. According to the prostatitis diagnosis guideline, prostatitis could be classified as acute bacterial prostatitis, chronic bacterial prostatitis, inflammatory prostatitis , noninflammatory prostatitis and asymptomatic prostatitis[11]. Prostatitis has a prevalence rate of 5 - 9% and accounts for over 2 million hospital visits annually in the USA[12]. Furthermore, many researchers and urologists believe that the incidence of asymptomatic prostatitis could be much higher than symptomatic prostatitis. This is supported by the fact that both inflammatory cells were found in the prostate biopsy, or leukocytes found in semen analysis from patients without a history of prostatitis[13]. The high prevalence of prostatitis could contribute to prostate carcinogenesis, which is the most common malignancy among elderly men in the United States, and the second most common cause of cancer-related death in males[14]. Currently, the confirmed risk factors for prostate cancer are: age, family history, and race [15,16]. However, this association between prostatitis and prostate cancer remains unclear, with studies containing both null and significant results. Roberts and his colleagues[17] conducted a study including cases and controls from Minnesota, and found there was a significant association between prostatitis and prostate cancer (OR = 1.7; 95% CI: 1.1-2.6). However, when he excluded cases of prostatitis within 2 years before the study ( most of them were acute prostatitis), the results turned out to be not significant (1.9; 0.9-3.8). This result is quite controversial, because chronic inflammation is identified with higher risk increasing cancer, compared with acute inflammation.

Meta analysis is a quantitative systematic method to test the effectiveness of exposure/treatment, in both cohort and case control studies. A previous meta analysis[18] involving 11 studies between1971-1996, provided statistical evidence that prostatitis is a significant risk factor in prostate cancer. Our investigation is based more on recent studies. This study is to involve recent studies related with prostatitis and prostate cancer. Moreover, in order to prevent other non-prostatitis disease confusing the analysis results, we set the Inclusion Criteria that all enrolled prostatitis cases in the studies must be diagnosed according to the National Institutes of Health (NIH) prostatitis guideline. This Inclusion Criteria could differentiate prostate inflammation disease from the other prostate disease, including cancer, benign prostate hyperplasia and so on[19].If there is sound evidence relating prostatitis and prostate cancer, it is possible to prevent, or treat prostate cancer by preventing prostatitis. This study is to provide epidemiological evidence to demonstrate the possible association between prostate cancer and prostatitis.

## Method

### Evidence acquisition

**Figure 1:**
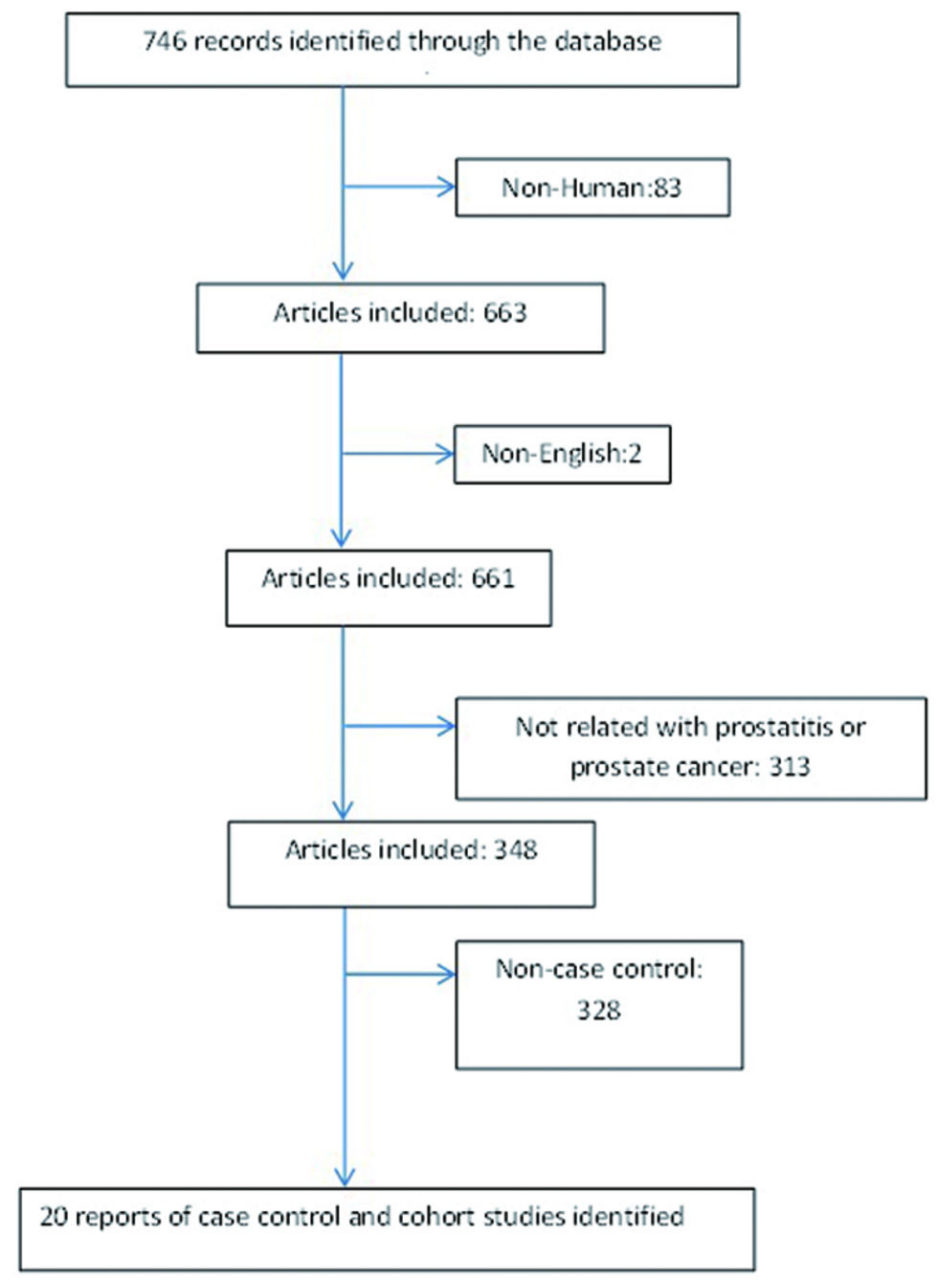
A flow diagram of the study selection process.

We conducted a literature search for ‘prostatitis’ and ‘prostate cancer’, or ‘inflammation’ and ‘prostate cancer’, or ‘meta’ and ‘prostatitis’ and ‘prostate cancer’ in both PubMed and Medline databases, based on English literature. In addition, the references and citations of studies were also reviewed. If cited or referenced articles were eligible, they would be included into pooled studies candidates in order to prevent the loss of any important and useful data. The selection criteria were as follows: human case control studies; published from May 1990 to July 2012; that contained number of prostatitis and prostate cancer cases. The study also must provide comprehensive information including: age, race/country, number of people with/without prostatitis, number of people with/without prostate cancer, study period, and data source (Clinical and non-clinical based). Additionally, in all qualified studies, the patients who were diagnosed and managed as prostatitis must meet the criteria for the NIH prostatitis guideline[19].In total, twenty case-control studies (5 clinical interview and 15 self-reports) were included in the final analysis(Figure 1)[11,20–39]. 

Quantitative Meta-analysis was performed using Stata 11. Data were imported under study name expressed by the last name of authors, year of study, country, race, data source, number of cases (cancer), number of controls, number of cases exposure (prostatitis) and number of unexposed cases not under exposure. One concern was that prostatitis has several classifications, including chronic bacterial prostatitis, acute prostatitis, and asymptomatic prostatitis. However, in our 20 studies, numbers of general prostatitis are given instead of numbers of different disease classification, and we are unable report related statistical results based on classification.

Odds ratios were calculated using the given information. Pooled log OR were calculated under a fixed effect model, which assume exposure’s effect on cancer cases is same across studies. Heterogeneity of ORs was tested. If p-value of heterogeneity was significant (<0.05), random effects model was more appropriate, which assumes that exposure’s effects on cancer cases are different across studies. Statistical methods in Meta-analysis are desired in the following literature review. In our study, we use both fixed effect and random effects model and provide related results in tables and figures.

### Statistical analysis

Stata version 11 (Stata Corp, TX, USA) was the main statistical tool used in this study. Both fixed effect and random effects model were used to test the effect of prostatitis in increasing the risk of prostate cancer. The fixed effect model assumes that the effect of treatment is the same among all studies, and the random effects model assumes that the effects might be different, and therefore, random effects model usually has a wider confidence interval. When heterogeneity is significant, it indicates that a random effects model is more appropriate than a fixed effect model.

Data were stratified based on the data source (clinical based case control or personal interview case control), country, and decades (1990’s, 2000’s, or 2010’s). All the stratification is tested to identify the bias and difference between different groups, based on the assumption of heterogeneity (fixed effect or random effects group). Publication bias was tested based on Egger’s test, to exam whether the bias is between the published and unpublished papers. Funnel plots were also graphed to check the small study effect. 

## Results

### Evidence synthesis

**Figure 2:**
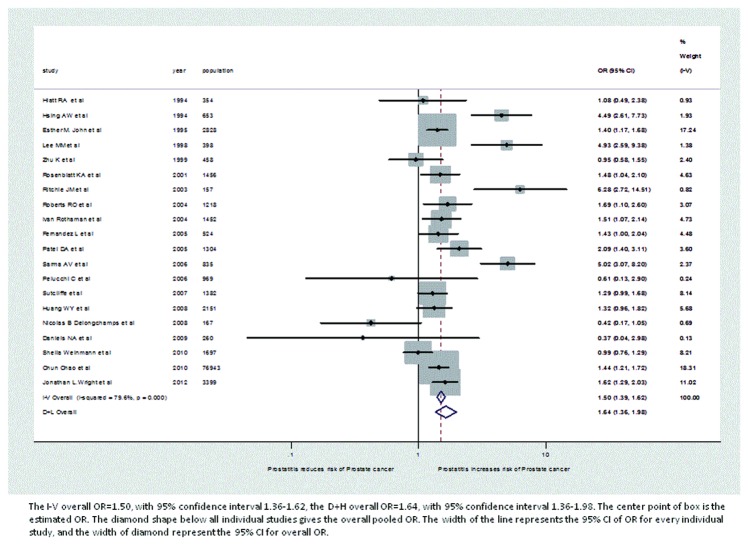
Forest plot of odds ratio under fixed effect and random effects model.

Table 1 shows the characteristics of included studies, including study design, number of subjects with each combination of case/control, exposed/unexposed status, race, age, and history of prostatitis and prostate cancer. Under the fixed effect model, the pooled OR=1.50. Under random effects model, the pooled OR=1.64. Forest plots are also provided to show the individual ORs in each study (Figure 2). In a forest plot, every individual study has a line with a box on it. The center point of the box is the estimated OR. The diamond shape below all individual studies gives the overall pooled OR. The gray blocks for each study indicate the different weights of the study, represented by the size of the block. The width of the line represents the 95% CI of OR for every individual study, and the width of diamond represents the 95% CI for the overall OR. In Figure 2, papers from Hsing AW[28], Lee MM[25] , Ritchie JM[11], and Sarma AV[29] have the highest ORs, but small weights. Furthermore, most of the studies with big weights, i.e., papers from Esther M. John[36], Chun Chao[39], and Jonathan L.Wright[37], also have ORs close to the estimated pooled ORs.

**Table 1:** Characteristics of studies of prostatitis and prostate cancer.

**Study**	**Year**	**Country**	**Race**	**Data source**	**No. Cancer cases**	**No. prostatitis in cases**	**No. controls**	**No. prostatitis in control**
**Hiatt RA et al.[24]**	**1994**	**U.S**	**White men and Black men, other**	**personal interview**	**177**	**14**	**177**	**13**
**Hsing AW et al.[28]**	**1994**	**China**	**Chinese**	**personal interview**	**115**	**28**	**538**	**36**
**Esther M. John et al.[36]**	**1995**	**U.S**	**black**	**personal interview**	**1642**	**414**	**1186**	**230**
**Lee MM et al.[25]**	**1998**	**China**	**Chinese**	**personal interview**	**133**	**32**	**265**	**16**
**Zhu K et al.[26]**	**1999**	**U.S**	**NA**	**clinical based**	**222**	**37**	**236**	**41**
**Rosenblatt KA et al.[22]**	**2001**	**U.S**	**White men and Black men**	**personal interview**	**753**	**87**	**703**	**57**
**Ritchie JM et al.[11]**	**2003**	**U.S**	**White , other**	**personal interview**	**58**	**24**	**99**	**10**
**Roberts RO et al.[20]**	**2004**	**U.S**	**NA**	**clinical based**	**409**	**41**	**809**	**50**
**Ivan Rothaman et al.[33]**	**2004**	**U.S**	**NA**	**personal interview**	**750**	**90**	**702**	**58**
**Fernandez L et al.[27]**	**2005**	**Cuba**	**White, black, other**	**personal interview**	**271**	**183**	**253**	**150**
**Patel DA et al.[31]**	**2005**	**U.S**	**black and white men**	**personal interview**	**700**	**86**	**604**	**38**
**Sarma AV et al.[29]**	**2006**	**U.S**	**African American**	**personal interview**	**129**	**34**	**706**	**47**
**Pelucchi C et al.[30]**	**2006**	**Italy**	**white**	**personal interview**	**280**	**2**	**689**	**8**
**Sutcliffe et al.[32]**	**2007**	**U.S**	**White black; Asian**	**personal interview**	**691**	**152**	**691**	**124**
**Nicolas B Delongchamps et al.[21]**	**2008**	**U.S**	**NA**	**clinical based**	**22**	**11**	**145**	**102**
**Huang WY et al.[35]**	**2008**	**U.S**	**White, black**	**personal interview**	**868**	**78**	**1283**	**89**
**Daniels NA et al.[23]**	**2009**	**U.S**	**Asian or Pacific Islander, Black, Latino or Hispanic, White**	**clinical based**	**65**	**1**	**195**	**8**
**Sheila Weinmann et al.[34]**	**2010**	**U.S**	**White, black**	**clinical based**	**768**	**119**	**929**	**145**
**Chun Chao et al.[39]**	**2010**	**U.S**	**White, black, Asian, Hispanic**	**personal interview**	**1559**	**139**	**75384**	**4788**
**Jonathan L.Wright et al.[37]**	**2012**	**U.S**	**white and Black**	**personal interview**	**1754**	**217**	**1645**	**132**

**Figure 3:**
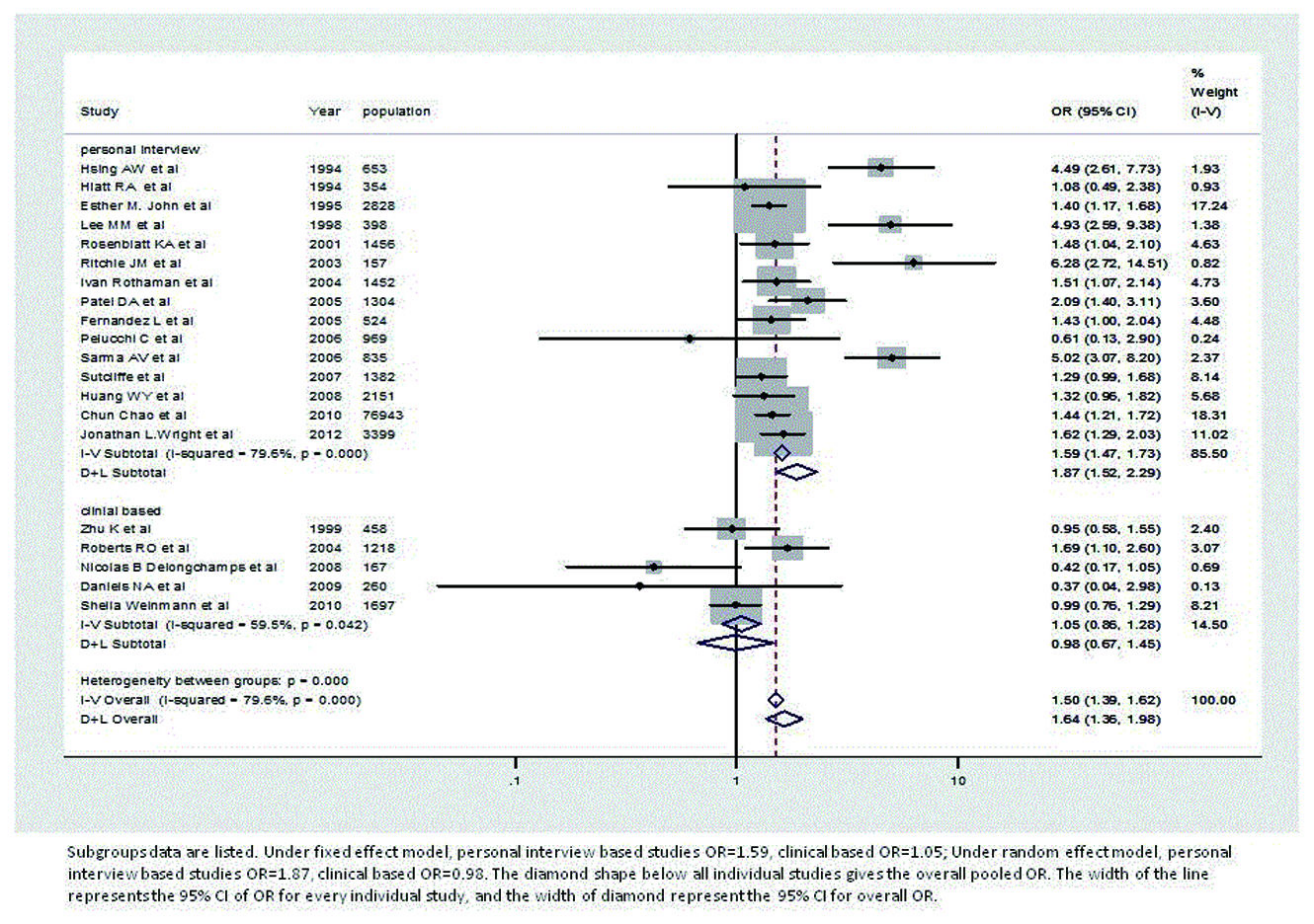
Forest plot of odds ratio under fixed effect and random effects model, based on data source.

Table 2 reports pooled OR for prostate cancer and prostatitis by study design. Under the fixed effect model, self-report based studies pooled OR=1.59, and clinical based studies OR=1.05. Under the random effects model, personal interview based studies pooled OR=1.87, and the clinical based studies OR=0.98. Forest plots are also given based on study types (see Figure 3). In Figure 3, all four papers with the largest ORs, and small weights are all included in self-report studies[11,25,28,29] as well as studies with larger weights, but smaller ORs[36,37,39]. In the clinical based interview, more studies with low ORs were included. In addition, there were 10 self-reported studies and 5 clinical based studies included. 

**Table 2:** Pooled Odds Ratio for prostate cancer and prostatitis by study design.

		**Fixed effect model**		**Heterogeneity**	**Random effects model**	
**study type**	**n**	**OR**	**95% CI**		**OR**	**95% CI**
**all**	**20**	**1.50**	**1.39-1.62**	**<0.001**	**1.64**	**1.36-1.98**
**personal interview**	**15**	**1.59**	**1.47-1.73**	**<0.001**	**1.87**	**1.52-2.29**
**clinical based**	**5**	**1.05**	**0.86-1.28**	**0.042**	**0.98**	**0.67-1.45**

**Figure 4:**
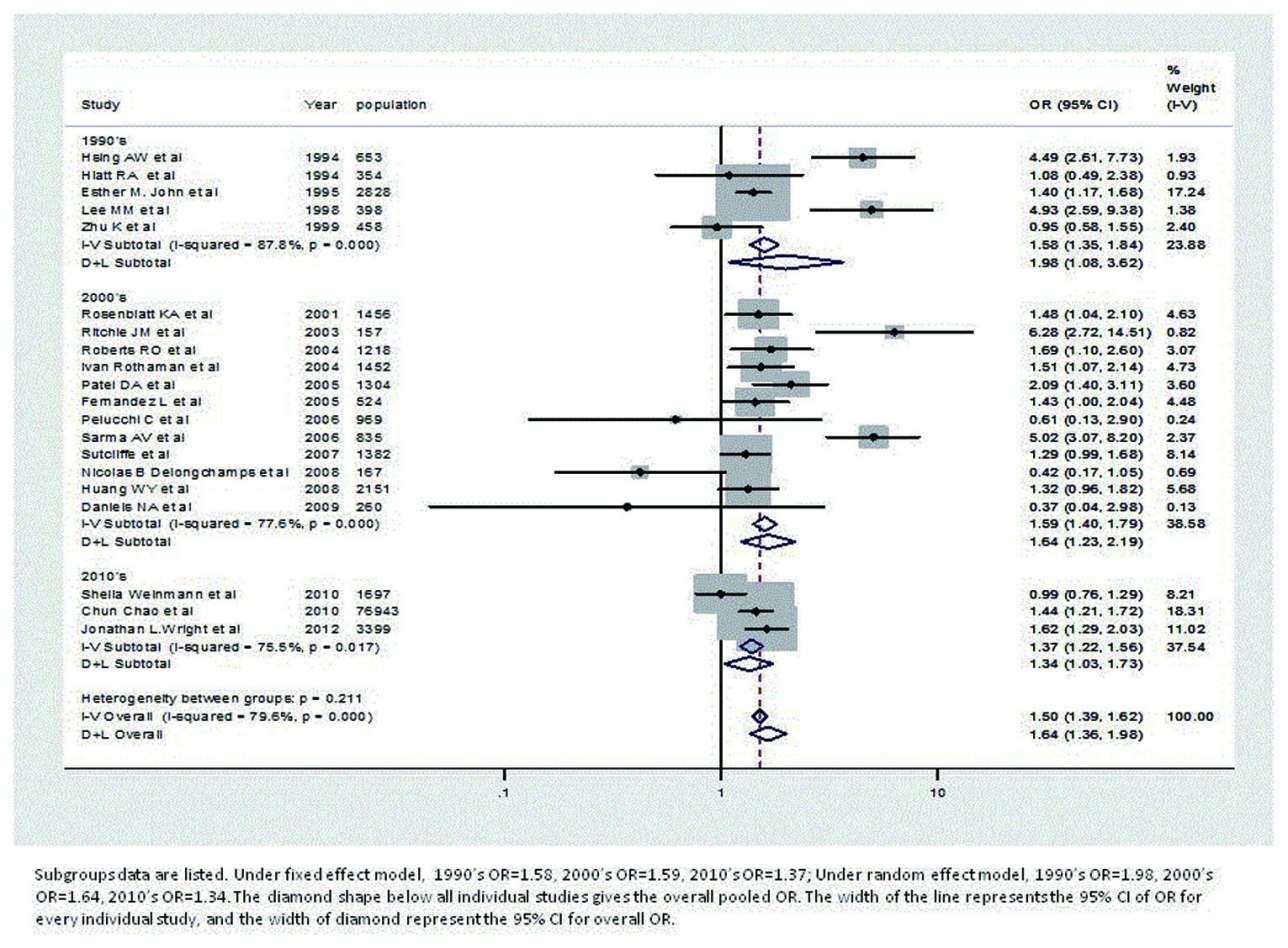
Forest plot of odds ratio under fixed effect and random effects model, based on decades.

Table 3 summaries the pooled ORs, based on decades, in both fixed effect model and random effects model. In all three decades analyzed (1990’s, 2000’s and 2010’s), the pooled ORs are all significant (both fixed effect model and the random effects model). Studies from 2000’s have the largest pooled log ORs, under the fixed effect model: 1.59, 95% CI: 1.40-1.79. Studies from 1990’s have the largest pooled log ORs, under the random effects model: 1.98, 95% CI: 1.08-3.62. The studies with lowest ORs are all included in2000’s [21,23,30], however, due to the small weights, these studies do not greatly affect the pooled log ORs. The number of studies is largest among those from 2000’s, while the number of studies in 2010’s is the smallest (see Figure 4).

**Table 3:** Pooled Odds Ratio for prostate cancer and prostatitis by decades.

		**Fixed effect model**		**Heterogeneity**	**Random effects model**	
**study type**	**n**	**OR**	**95% CI**		**OR**	**95% CI**
**All**	**20**	**1.50**	**1.39-1.62**	**<0.001**	**1.64**	**1.36-1.98**
**1990's**	**5**	**1.58**	**1.35-1.84**	**<0.001**	**1.98**	**1.08-3.62**
**2000's**	**12**	**1.59**	**1.40-1.79**	**<0.001**	**1.64**	**1.23-2.19**
**2010's**	**3**	**1.37**	**1.22-1.56**	**0.017**	**1.34**	**1.03-1.73**

**Figure 5:**
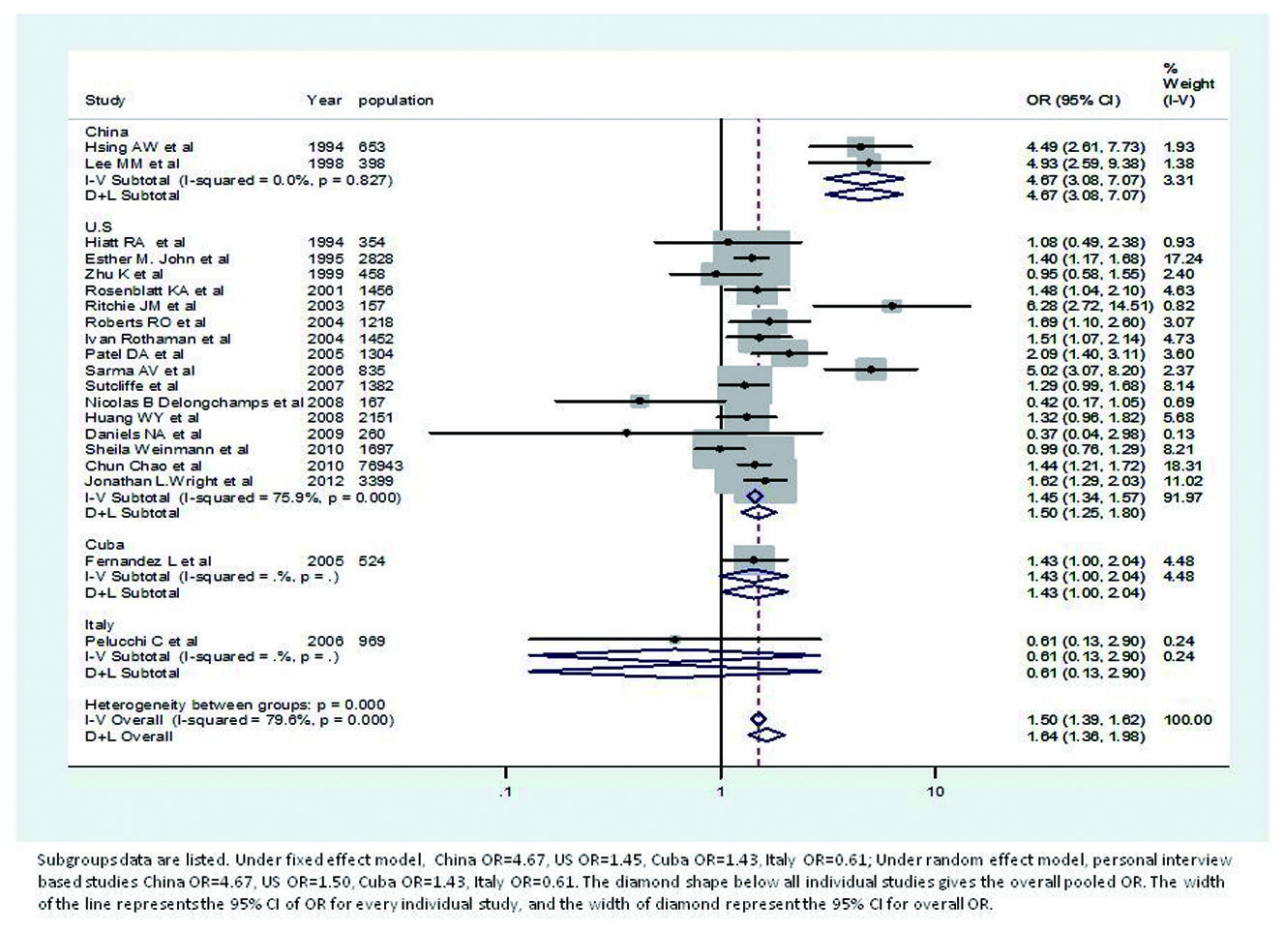
Forest plot of odds ratio under fixed effect and random effects model, based on countries.

Table 4 reports the ORs based on stratification of countries, in both fixed effect model. Forest plots of studies from China have the highest pooled log ORs, under both the fixed effect model and the random effects model, while the number of studies included in China is the smallest (Figure.5). When analyzed according to country, studies from Italy and Cuba do not have significant ORs. Finally, the number of studies included in U.S is the largest, and the pooled log ORs are quite close to the overall pooled log ORs. 

**Table 4:** Pooled Odds Ratio for prostate cancer and prostatitis by countries.

		**Fixed effect model**		**Heterogeneity**	**Random effects model**	
**study type**	**n**	**OR**	**95% CI**		**OR**	**95% CI**
**All**	**20**	**1.5**	**1.39-1.62**	**<0.001**	**1.64**	**1.36-1.98**
**U.S**	**16**	**1.45**	**1.34-1.57**	**<0.001**	**1.50**	**1.25-1.80**
**China**	**2**	**4.67**	**3.08-7.07**	**0.827**	**4.67**	**3.08-7.07**
**Cuba**	**1**	**1.43**	**1.00-2.04**	**.**	**1.43**	**1.00-2.04**
**Italy**	**1**	**0.61**	**0.13-2.90**	**.**	**0.61**	**0.13-2.90**

### Publication bias and small study effect

**Figure 6:**
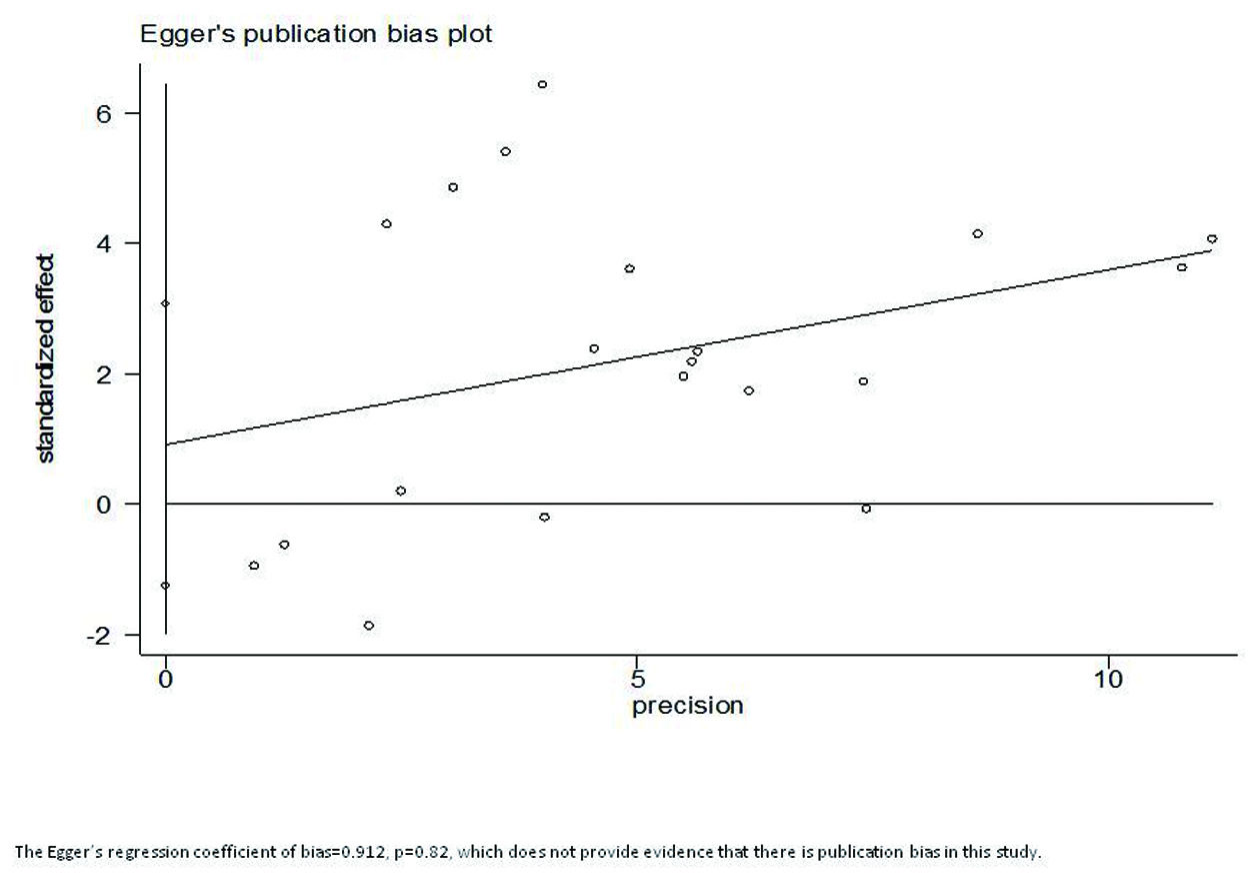
Egger’s publication bias plot.

Figure 6 reports Egger’s publication bias results, with p-value=0.82>0.05, and the intercept is 0.91. This insignificant p-value indicates that bias (intercept) is not significantly different from ‘0’, thus, there is not enough evidence to conclude that there is publication bias in this study. 

**Figure 7:**
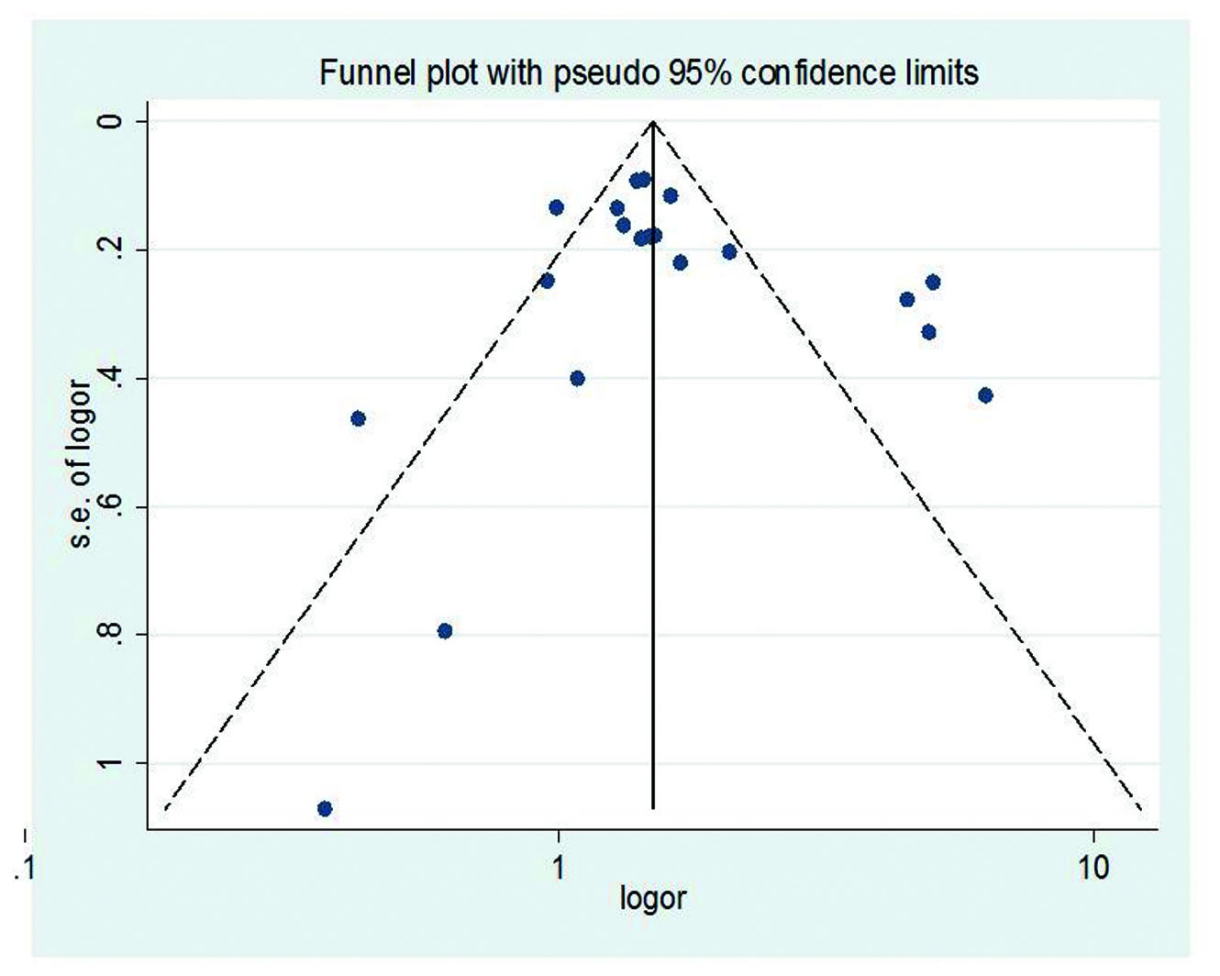
Funnel plot of standard error of log OR over log OR.

In Figure 7, most studies are at the top of funnel, with a large sample size. Some studies with smaller sample sizes were spread across the bottom of funnel. However, most studies are at the dashed bonds, which represent a lack of bias and heterogeneity, 95% of studies are expected to lie in this triangle area. The funnel is symmetric to the middle line, which is from the top of the triangle; also indicate no evidence of small study effects.

## Discussion

This study demonstrates that there is a significant positive relationship between prostatitis and prostate cancer, under both fixed effect and random effects model. This conclusion agrees with many previous biological and epidemiological studies mentioned above [4,6–10], which indicate that the inflammatory mediators could promote the prostatic carcinogenesis via multiple signaling pathways. Some examples include: inhibiting apoptosis, promoting cell proliferation, and inducing tumor suppressor gene loss. Our statistic of heterogeneity (p<0.001) indicates that the random effects model, which assumes that prostatitis has different effects on prostate cancer across studies, is more appropriate. However, the lack of cohort studies makes it difficult to conclude that there is a causal relationship between prostatitis and prostate cancer. 

From Table 1, overall OR (fixed: pooled OR=1.5, random: pooled OR=1.63) demonstrates that there is a significant effect of prostatitis on prostate cancer, but the OR in different strata is not always significant. When data is stratified by country for both the fixed and random effects model, studies from the U.S and China have significant pooled ORs (p<0.001, see Appendix), while studies from Cuba (fixed: p=0.057, random: p=0.057, see Appendix) and Italy (fixed: p=0.559, random: p=0.559, see Appendix) did not have significant results. One possible explanation is that there is not sufficient published evidence representing Cuban (n=1) and Italian (n=1) nationalities. We suspect that the lack of evidence is due to the language barrier, since we only included articles written in English. Studies with small sample sizes like the ones from Cuba and Italy may result in increased variation between people in the case and control groups, thus making it less likely for them to produce significant evidence or conclusions. This result could also be reflected from the forest plots. When data is stratified based on data source, clinical based and personal interview, the overall pooled ORs (fixed: p<0.001, random: p<0.001, see Appendix) are significant. Alone, personal interview group’s pooled ORs (fixed: p<0.001, random: p<0.001) are significant in both the fixed and random effects model. Clinical based studies (fixed: p=0.678, random: p=0.915, see Appendix) are both insignificant across the fixed and random effects groups. One possible explanation for the insignificant results among clinical based studies might be the small number of studies (n=5). Another possibility is the existence of both recall bias and detection bias among controls and cancer cases in the clinical based studies. Controls in clinical based groups may over-recall their previous medical history. For example, a patient may believe that, prostatitis might be related to their current health problems, effectively obscuring the difference between prostatitis and prostate cancer. Similarly, some kinds of prostatitis are undetected and asymptomatic, which do not express physical symptoms, i.e. pain, inflammation, urinary tract infection, and therefore, rates of prostatitis among cancer cases might be underestimated[40,41]. Recall bias and detection bias may also happen in non-clinical based groups, and will also affect the recording rate of prostatitis.

Publication bias and small study effect were also considered in this study. According to Egger’s publication bias plot, there was no evidence that unpublished papers and studies have significant effects on this study (p=0.820), with the intercept=0.912. Similar results were obtained when using Begg’s Funnel plot, where small studies are widely spread at the bottom of funnel. Thus, funnel plots are almost symmetric, and no small study effects observed. 

There are some limitations of this study. First, due to restriction placed on language and datasets, there are more studies, which were not included in this study, causing a potential bias. In addition, unpublished papers, especially ones without significant results, may still have effects on the overall pooled OR. Second, prostatitis is a clinically progressive (acute prostatitis can become chronic prostatitis), multi-classification disease, according to the prostatitis diagnosis guideline. Most of the studies we considered did not divide the prostatitis cases into different classification, and that may be a problem if all types of prostatitis are related to prostate cancer. Prostate cancer is also associated with age and race. However due to limited numbers of cases and controls within each age and race stratum, this study failed to find a relationship between age, race. 

## Supporting Information

Checklist S1 **Prisma checklist.**
(DOC)
